# Investigation of microstructure in additive manufactured Inconel 625 by spatially resolved neutron transmission spectroscopy

**DOI:** 10.1080/14686996.2016.1190261

**Published:** 2016-07-08

**Authors:** Anton S. Tremsin, Yan Gao, Laura C. Dial, Francesco Grazzi, Takenao Shinohara

**Affiliations:** ^a^ Space Sciences Laboratory, University of California at Berkeley, Berkeley, CA, USA; ^b^ General Electric Global Research Center, Niskayuna, NY, USA; ^c^ Consiglio Nazionale delle Ricerche, Istituto Sistemi Complessi (CNR-ISC), Sesto Fiorentino (FI), Italy; ^d^ Japan Atomic Energy Agency Tokai-mura, Naka-gun Ibaraki, Japan

**Keywords:** Non-destructive testing, additive manufacturing, microstructure, neutron imaging, 10 Engineering and Structural materials, 106 Metallic materials, 303 Mechanical/Physical processing, 504 X-ray/Neutron diffraction and scattering

## Abstract

Non-destructive testing techniques based on neutron imaging and diffraction can provide information on the internal structure of relatively thick metal samples (up to several cm), which are opaque to other conventional non-destructive methods. Spatially resolved neutron transmission spectroscopy is an extension of traditional neutron radiography, where multiple images are acquired simultaneously, each corresponding to a narrow range of energy. The analysis of transmission spectra enables studies of bulk microstructures at the spatial resolution comparable to the detector pixel. In this study we demonstrate the possibility of imaging (with ~100 μm resolution) distribution of some microstructure properties, such as residual strain, texture, voids and impurities in Inconel 625 samples manufactured with an additive manufacturing method called direct metal laser melting (DMLM). Although this imaging technique can be implemented only in a few large-scale facilities, it can be a valuable tool for optimization of additive manufacturing techniques and materials and for correlating bulk microstructure properties to manufacturing process parameters. In addition, the experimental strain distribution can help validate finite element models which many industries use to predict the residual stress distributions in additive manufactured components.

## Graphical abstract

**Figure:**
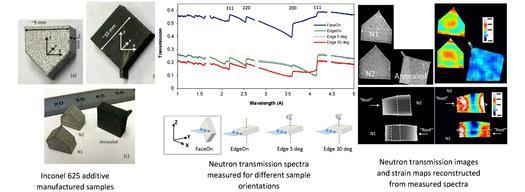

## 1. Introduction

The additive manufacturing (AM) technology recently progressed from the technique mostly used for rapid prototyping to production of complicated engineering components consisting not only of plastics but also of metals and metallic alloys [1–4]. In many cases additive manufacturing can produce parts that cannot be made with conventional machining technology. Additive manufacturing is fundamentally different than many traditional, subtractive manufacturing processes. In direct metal laser melting (DMLM) or selective laser melting (SLM) processes, substantial differences in microstructure are observed as compared to traditional manufacturing methods. In these processes where samples are grown layer by layer, the microstructure is shaped by the interaction of materials within the layer and between subsequent layers as complex thermal processing cycles are applied. Residual stress distribution is among the most important microstructural variables to be investigated and understood by additive manufacturers in the industry. Also the presence of processing defects (e.g. micropores or impurities) can introduce local strength deficiencies. Despite all the knowledge and optimization of the manufacturing tools there is a substantial variability between different machines.[4] At present, additive manufactured components may still be inferior to conventionally fabricated ones in overall performance. Therefore understanding the links between the microstructure, composition, and processing parameters is very important for the progress of this technology.[1–5]

In this article we demonstrate the possibility of studying the microstructure of Inconel 625 materials made by additive manufacturing with energy-resolved neutron imaging. This neutron technique can provide microstructure and composition information on relatively large components, which cannot be obtained by conventional characterization techniques, such as optical and electron microscopies, and X-ray diffraction. The unique capability of neutrons to penetrate thick metal samples (e.g. several centimeters of steel, titanium and nickel-based materials) can reveal the bulk properties of samples without alteration of sample integrity. Conventional neutron radiography and tomography are widely used to reveal the internal structure of complicated objects.[6,7] including additive manufacturing samples.[8] Addition of spectroscopic information in each pixel of the detector [9,10] also enables studies of material microstructures, such as texture,[11–14] strain,[15–17] grain orientation,[18] and, in some cases, bulk elemental composition.[19,20] This paper presents the results of the non-destructive study of Inconel 625 samples, manufactured by the DMLM method.[1] The results of these experiments demonstrate the capability of achieving ~100 μm spatial resolution for investigating the bulk microstructure properties of metal AM samples.

## 2. Methods

**Figure 1.**
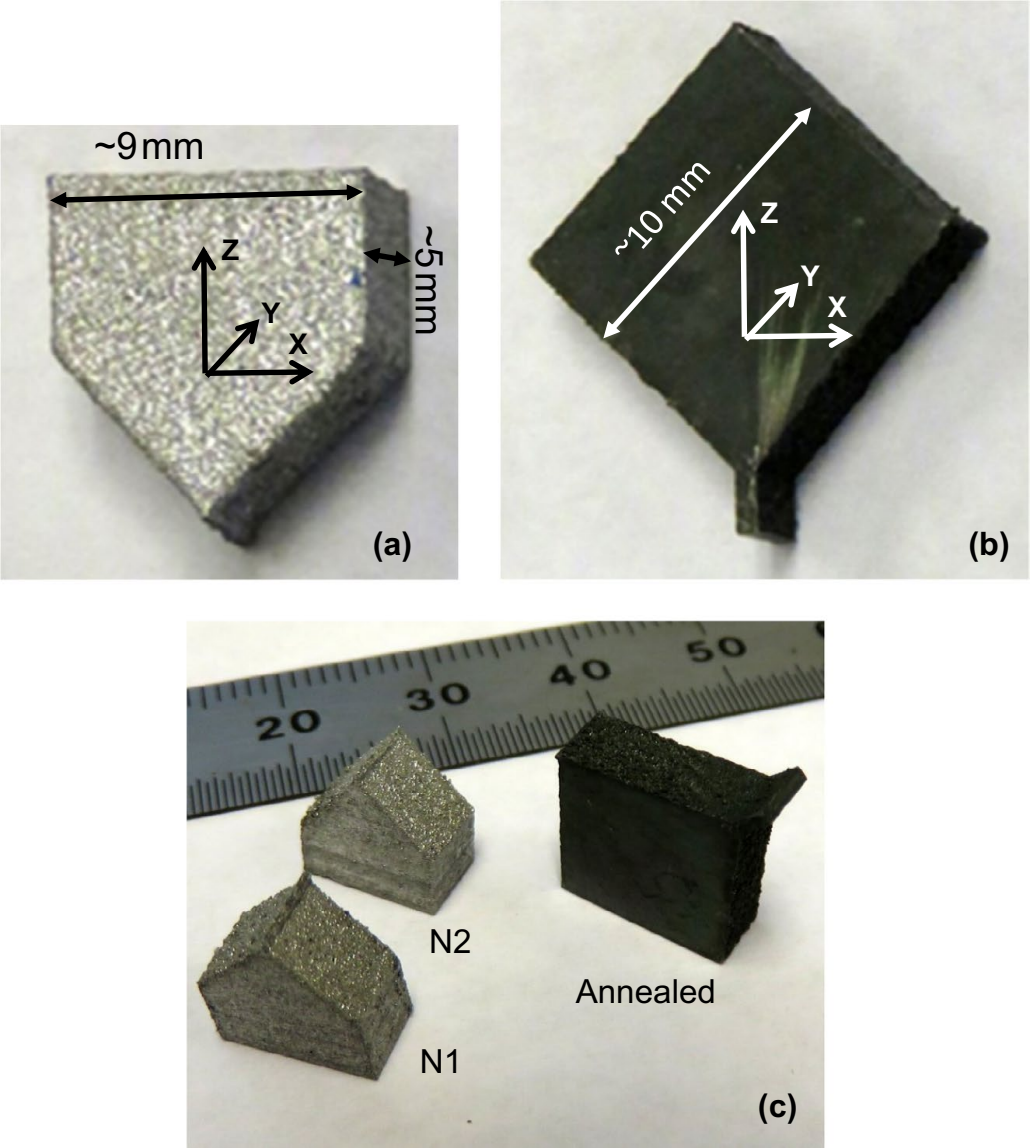
Photograph of Inconel 625 additive manufacturing samples investigated in the present study. The samples were measured in two orthogonal orientations: with neutrons propagating along the Y-axis and along the X-axis. The sample build direction was along the Z-axis. The sample shown in (b) was annealed for 2 h in argon at 1050°C.

The samples studied in this experiment were manufactured by using an SLM 250 additive manufacturing machine (SLM Solutions GmbH, Lübeck, Germany). Two 9 × 9 × 5 mm samples, N1 and N2, were prepared using similar processing parameters (Figure 1). A third additional sample (as shown in Figure 1(c)) was built in the same way and was heat treated at 1050°C in argon for 2 h and air cooled prior to measurement to relieve as-built stresses in the part. This sample was used as an unstrained reference for strain calculation as described below. The photographs of the samples are shown in Figure 1, with growth direction along the Z-axis. During the experiment, neutrons were propagated along both the Y-axis (referred to as face-on orientation) and X-axis (edge-on orientation). There are several methods to measure spatially resolved neutron transmission spectrum. Velocity selectors [21] and double crystal monochromators [22,23] can be utilized at continuous neutron sources. The energy resolution ΔE/E in these setups is ~10–15% for the former and ~3% for the latter. The transmission spectra in these experiments can be taken by scanning the neutron energy and recording consecutive images at each energy. In some cases that energy resolution is sufficient for the studies of microstructure in metal samples, where the presence of Bragg edge scattering provides the contrast in the transmission images. However, measurement of strain is limited by the energy resolution of these scanning techniques. It has been demonstrated that imaging of strain distribution can be done with better accuracy, down to ~100 microstrain [15–17] if neutron spectra are measured with 10× better energy resolution of ~0.3%, as in the case of pulsed neutron sources. It is important to note that the neuron transmission spectrum over a broad energy range can be obtained simultaneously in one measurement at pulsed neutron sources. Although studies involving measurements of neutron transmission spectra at spallation sources have been widely used to date in a single pixel configuration, it is only recently that spatially resolved neutron transmission has been possible due to the development of event counting neutron detectors with multiple pixels, such as 10 × 10 detector with 2 × 2 mm^2^ pixels [24], 512 × 512 detector with 55 × 55 μm^2^ pixels [25,26] and event-encoding detectors [27,28] with sub-mm spatial resolution.

**Figure 2.**
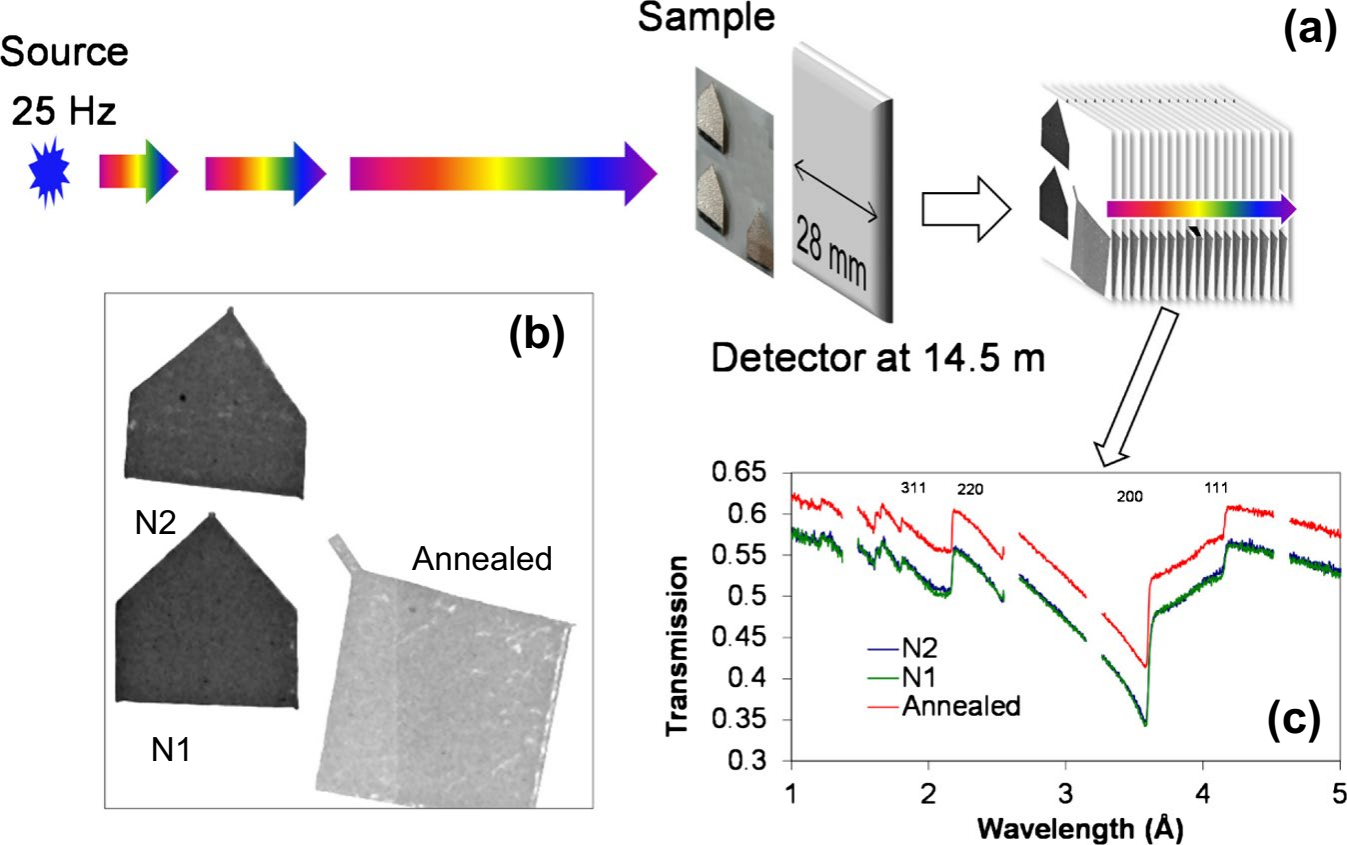
(a) Schematic diagram of measurement setup: neutrons with a wide energy range are produced by a pulsed source. Sample is placed in front of detector at 14.5 m distance from the source. A set of images (several thousands), each corresponding to a certain range of neutron energy/wavelength is acquired simultaneously. The full spectrum (summed over the entire energy range) transmission image is shown in (b). Transmission spectra (c) can be obtained from selected areas of the image.

The experiments reported in this paper were conducted at the Noboru beamline [29] of Japanese Spallation Neutron Source, operated at 25 Hz frequency and 250 kW power during the measurements. The neutron pulses are produced by spallation initiated by two 100 ns proton pulses, separated by 600 ns. After moderation the spallation neutrons propagated towards the sample and detector over ~14.5 m distance, Figure 2. The energy of each neutron can be calculated from its time of flight (TOF) providing the detection system can record the time of neutron arrival relative to the source trigger. A neutron counting MCP/Timepix detector [25,26] was used for the measurement of the transmission spectra in each pixel. Unique neutron sensitive microchannel plates for this detector were provided by Nova Scientific, Inc (Sturbridge, MA, USA). This type of detector is capable of registering multiple (>10^5^) simultaneous events with 55 μm spatial resolution and 20–500 ns timing resolution, depending on the energy of the neutron. A dataset consisting of several thousand images, each corresponding to a particular neutron energy, was acquired in each measurement. Thus 262144 spectra, from the detector consisting of 512 × 512 pixels, were acquired simultaneously, enabling spatially resolved studies of bulk microstructure. The data were acquired with the energy binning of ΔE/E ~0.05–0.1%. The strain was reconstructed from datasets integrated over several hours, in order to acquire sufficient number of neutron counts in each energy bin and at each pixel of the 2D detector.

### 2.1. Reconstruction of strain distribution across the samples

The high degree of penetration into many metallic materials makes neutrons unique probes of internal microstructure in these samples, which can be as thick as a few centimeters (and even tens of centimeters for the case of aluminum). Measurements of neutron transmission spectra in each 55 μm pixel, enabled recently by advances in neutron instrumentation, allow the analysis of microstructure with a high spatial resolution. In the transmission configuration the measured spectra apparently provides information on the microstructure averaged over the entire sample thickness, except for some special cases such as imaging of a diffracted signal from a single crystal or grain, which can be specific to a certain crystal/grain and is independent from the surrounding materials. Therefore the stain reconstructed in the present study represents the value averaged along the path of neutrons within the sample. However, that averaged strain value can be reconstructed within each 55 μm pixel, providing there is sufficient number of neutrons acquired in such a small area and maps of residual strain distribution can be obtained across the entire sample in one measurement.

The presence of sharp variation in transmission spectra, often referred to as Bragg edges, caused by diffraction of incident neutrons, enables determination of crystal lattice strain, as suggested in [30] and demonstrated in subsequent experimental studies.[15–17] The wavelength *λ*_*hkl*_ at which neutrons scatter to angle *ϑ* from a set of planes with interplanar distance *d*_*hkl*_ is determined by the Bragg equation:

(1) $$\lambda_{hkl}=2d_{hkl\;}\sin\vartheta$$

where the Miller indices (*hkl*) [31] define a specific set of lattice planes in a crystalline sample. With ϑ reaching π/2 neutrons are scattered backward and there is no scattering possible at longer wavelength for that set of planes, which results in a sharp increase of neutron transmission. Thus measurement of a Bragg edge wavelength is directly related to the interplanar distance *d*_*hkl*_ and can be used for determination of strain from the equation:

(2) $$\varepsilon =\left(d-d_{0}\right)/d_{0}=\left(\lambda -\lambda_{0}\right)/\lambda_{0}.$$

where *d*_0_ is the interplanar distance of the unstrained material. In this study we assumed that the strains were relieved in the annealed sample and consequently the averaged value of *d*_*hkl*_ of that sample was taken as the unstrained interplanar distance *d*_0_ for a particular Bragg edge.

The energy resolution of our experimental setup is determined by the width of the moderated neutron pulse, typically ΔE/E ~ 0.2–0.4% for the wavelengths of interest, limiting reconstructed strain values to 2000–4000 microstrain resolution. However, the position of the Bragg edge, as in case of diffraction techniques, can be determined with a better accuracy by fitting an analytical function to measured spectra,[15–17,30] improving the resolution to ~100 microstrain. The fitting of an analytical function with five fitted parameters (*λ*_*e*_ – the wavelength of the Bragg edge; *σ*, *τ*, *C*_1_ and *C*_2_ – the width, asymmetry, offset and height of the edge, respectively) [15,16,30]

(3) $$T({\left.\lambda )\right|}_{\lambda_{0},\sigma ,\tau ,C_{1},C_{2}}=C_{1}+C_{2}\left[erfc\left(\frac{\lambda_{e}-\lambda }{\sqrt{2}\sigma }\right)-\exp\left(\frac{\lambda_{e}-\lambda }{\tau }+\frac{\sigma^{2}}{2\tau^{2}}\right)*erfc\left(\frac{\lambda_{e}-\lambda }{\sqrt{2}\sigma }+\frac{\sigma }{\sqrt{2}\tau }\right)\right]$$

can be performed for the spectra acquired for each 55 × 55 μm^2^ pixel of the dataset. However, due to the limited neutron flux, the measured spectrum in such a small area is noisy even after several hours acquisition time. In order to reduce the statistical noise we combined the spectra from neighboring pixels, typically in the area from 0.5 × 0.5 to 1.5 × 1.5 mm^2^, and repeated that process ~250,000 times covering all 55 μm pixels of the dataset. The spectral averaging obviously would smooth sharp variations of reconstructed strain with “running average”, but still allows for the averaged strain reconstruction within each 55 μm pixel.

## 3. Results

### 3.1. Full spectrum radiography

**Figure 3.**
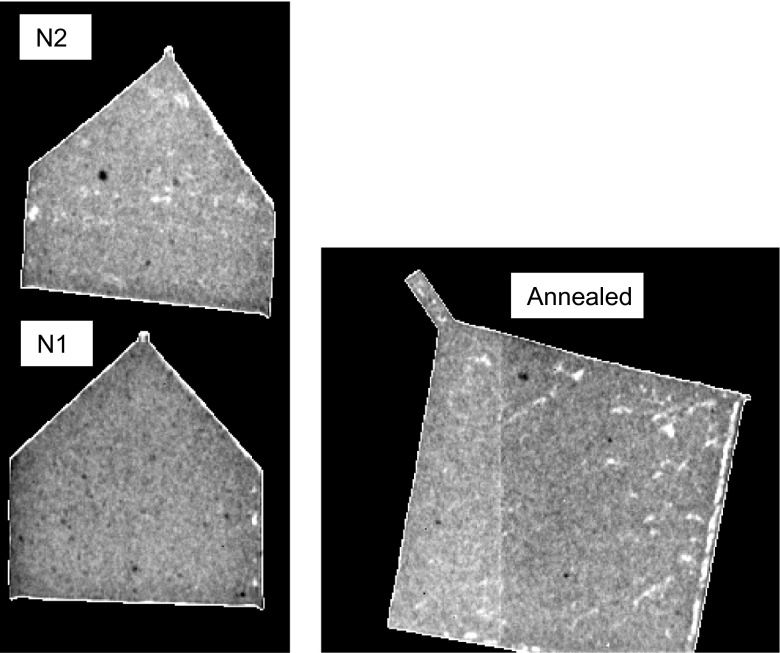
Neutron transmission images obtained by summation over the entire energy range (full spectrum transmission). The features seen within the samples are due to the presence of inclusions or voids, as confirmed by the images shown in Figure 7. The vertical line seen in the annealed sample is an artifact of detector calibration (the border between the two chips of the readout is visible).

The conventional white-spectrum neutron radiography (summation over all images in a dataset) of the samples is shown in Figure 3. The contrast in these images is due to the density and/or sample composition variation instead of crystallographic (including texture) differences. The white spots in these images are most likely due to voids and micropores, which in the case of the annealed sample are elongated perpendicular to the growth axis. The black spots are likely caused by impurities containing elements with larger neutron attenuation. The contrast in these images is greatly enhanced so that non-uniformities, even at a very small fraction of the sample volume, can be observed. No substantial difference between the annealed and the as-printed samples is observed based on the results shown in Figure 3.

### 3.2. Energy resolved neutron imaging

**Figure 4.**
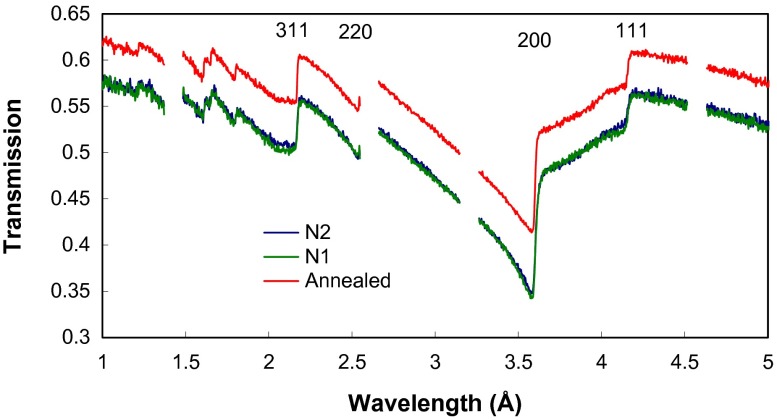
The measured transmission spectra of three samples with neutron beam along the Y-axis of the sample. Sharp increase of transmission is due to Bragg scattering at the corresponding crystallographic planes, marked with Miller indices. The gaps in the spectrum are due to the detector deadtime required for the data readout.

**Figure 5.**
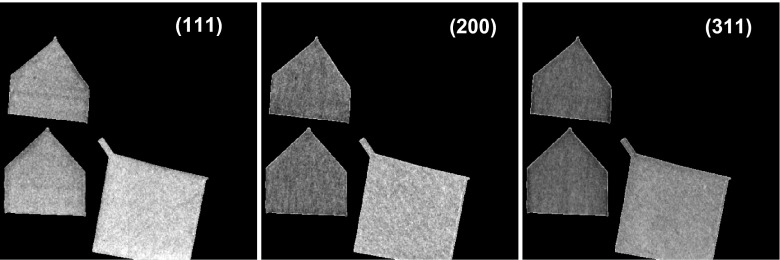
Neutron transmission images obtained at the narrow range of wavelength (0.1–0.2 Å) near the specific Bragg edges. The contrast is mostly due to the variation of crystallographic properties of the samples, e.g. texture.

**Figure 6.**
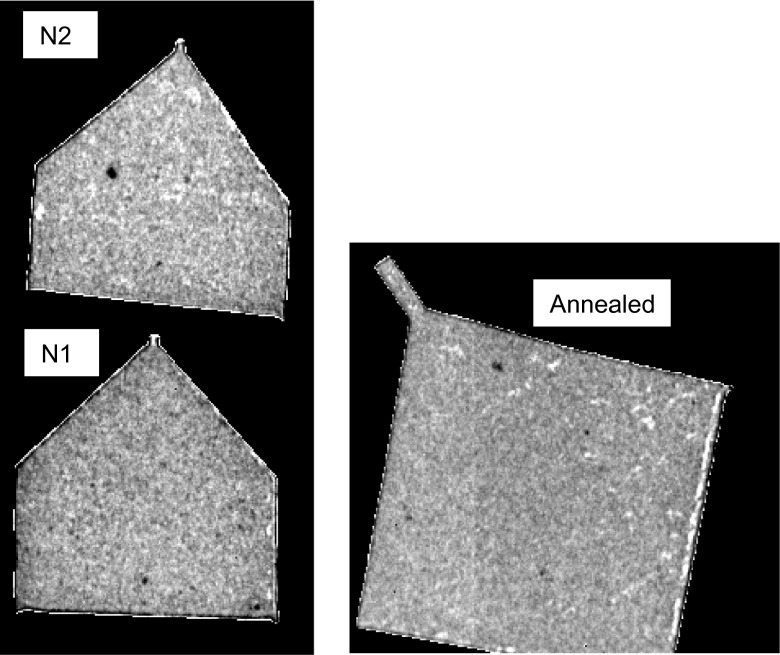
Neutron transmission image obtained by summation of images acquired for the wavelengths past the last Bragg edge (4.5 to 7.5 Å). The contrast is due to density/composition variation from either inclusions or voids, instead of crystallographic properties as no coherent scattering is possible at these wavelengths.

The energy-resolved imaging data enables extraction of the transmission spectra from small areas within a sample. The transmission spectra shown in Figure 4 exhibit sharp variations of transmission at a specific set of wavelengths, characteristic of the fcc crystal structure of Inconel 625. The transmission of the annealed sample is higher due to the smaller thickness of the sample along the Y-axis. There is no Bragg scattering above ~4.14 Å (the (1 1 1) edge wavelength), corresponding to the largest interplanar distance of ~2.07 Å in these samples. The images acquired in a narrow-energy range around each Bragg edge are shown in Figure 5. There are some weak features along the X-axis, in the growth plane, on the image around (1 1 1) edge, while the image at (2 0 0) edge reveals the vertical stripes, which are parallel to the growth direction. All these features are most likely related to the difference in texture or anisotropy of grain orientation. The lack of large contrast variation in these images indicates that these samples are uniform in texture and no gross crystal structural defects are present. The transmission image acquired with wavelengths above the last Bragg edge (4.5 to 7.5 Å, Figure 6) reveals the presence of features most likely caused by impurities and voids and not related to crystallographic properties as no scattering exists at these wavelengths.

### 3.3. Strain and microstructure along Y-axis reconstructed by Bragg edge analysis

**Figure 7.**
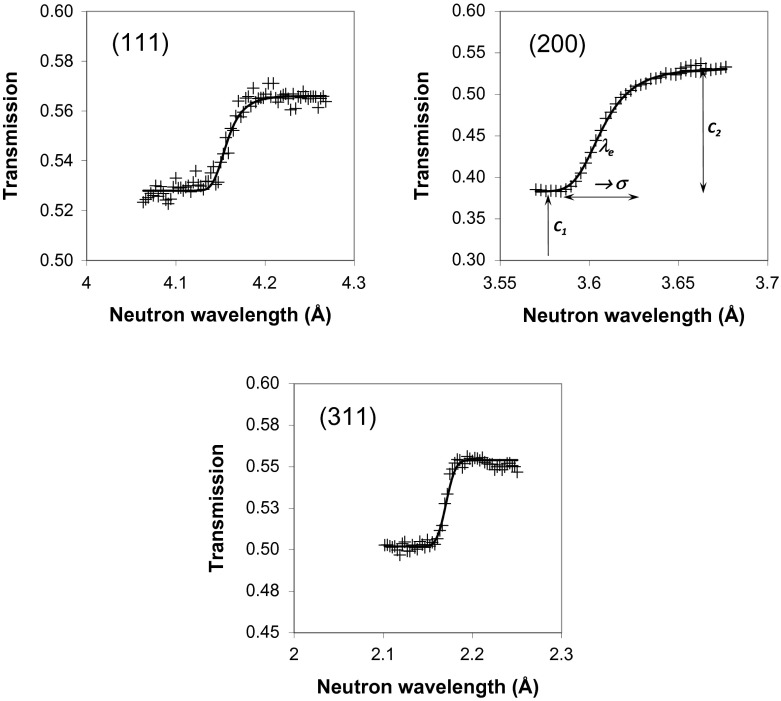
The measured Bragg edges for sample N1 (markers) and fitted curves. The spectra are from pixels summed over an area of several mm wide to improve counting statistics.

**Figure 8.**
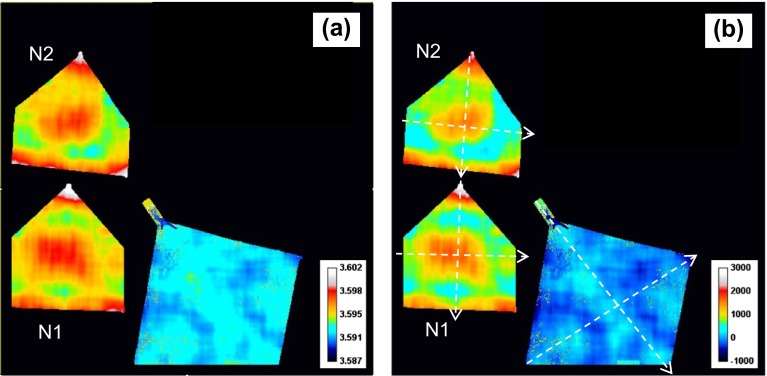
(a) Map of (2 0 0) Bragg edge wavelengths *λ*_*e*_ for the transmission spectra acquired along the direction perpendicular to the face of the samples (along the Y-axis as shown in Figure 1). *λ*_*e*_ values are obtained by fitting Equation (3) to the measured transmission spectra. Around 60,000 fits are performed for that image. The value in each pixel is based on fitting of combined spectra from a 30 × 30 pixels area (or 1.65 × 1.65 mm) around this pixel. The color scale indicates the values of the *λ*_*e*_ edge position. (b) Map of strain values (integrated along the Y-axis) calculated from (a) assuming the unstrained value *λ*_0_ of 3.592 Å (average value measured from the annealed sample). The color scale indicates strain values in microstrain. The dashed lines indicate the position of cross sections through the strain map shown in Figure 10.

**Figure 9.**
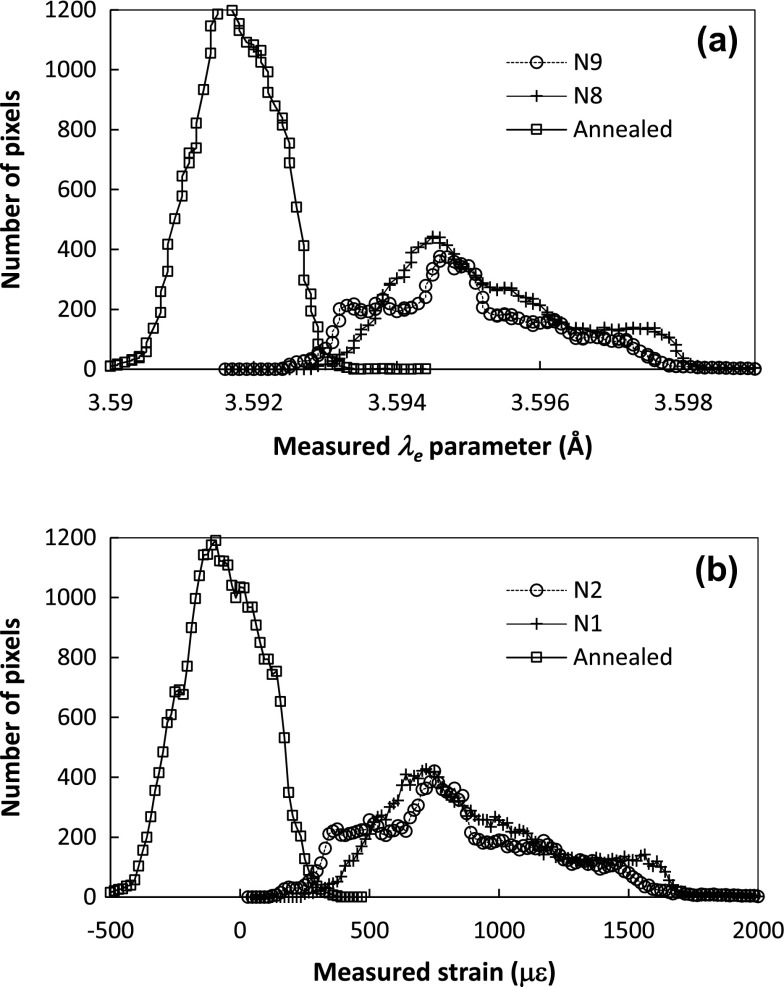
Histograms of measured *λ*_*e*_ parameter (2× of the interplanar lattice spacing) and strain at (2 0 0) Bragg edge: (a) distribution of measured *λ*_*e*_ values; (b) distribution of the strain values along the Y-axis.

**Figure 10.**
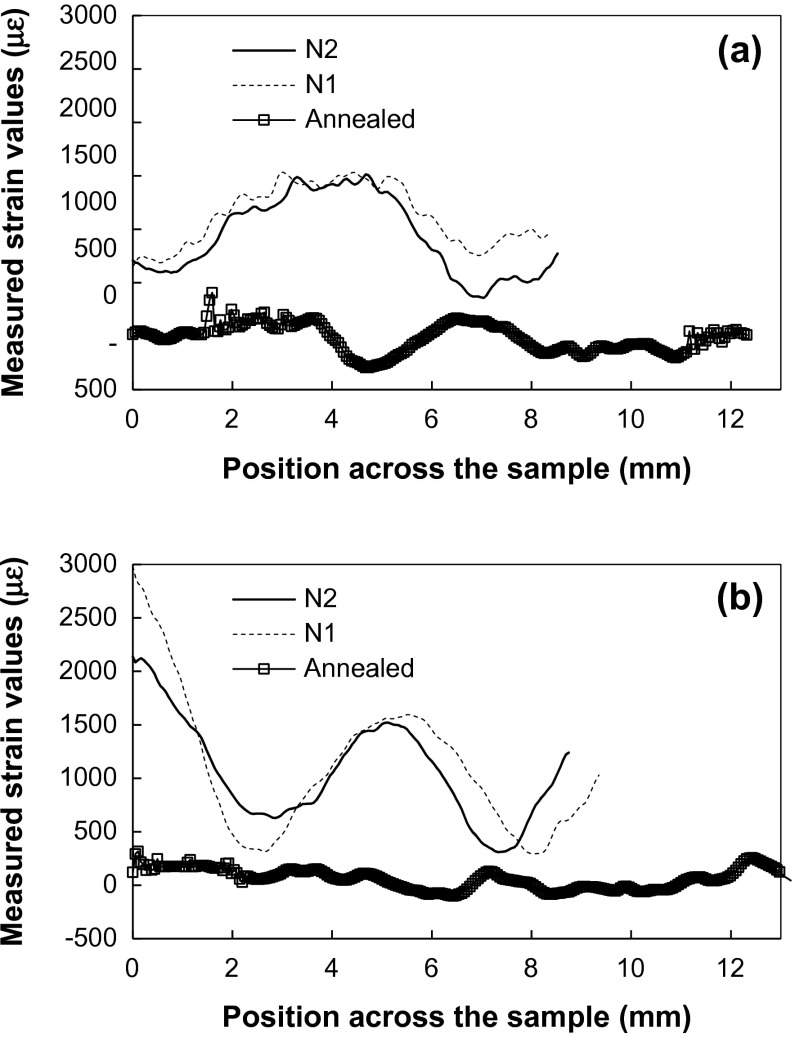
Cross sections (55 μm –wide) through the strain maps of three samples calculated for (2 0 0) Bragg edge shown in Figure 8(b). The dashed lines of Figure 8(b) indicate the position of cross sections in the samples. (a) Cross sections parallel to the growth layers (along the X-axis), (b) cross sections along the sample built direction (along the Z-axis).

The three Bragg edges used in the reconstruction of strain and texture are shown in Figure 7. The strain was calculated from Equation (2) for the measured edge wavelength, parameter *λ*_*e*_ in Equation (3), while variation of texture was reconstructed from parameter *σ* (the width), *C*_1_ (the offset) and *C*_2_ (the height) of the Bragg edges obtained by fitting to the spectra acquired around each pixel according to Equation (3). These reconstructed parameters obviously represent the values averaged over the sample thickness as measured transmission spectra is affected by absorption and scattering along the neutron path within the entire sample. The noise of measured data for (1 1 1) edge is the highest among three edges due to the amplitude of that edge being the smallest, while the strongest edge was (2 0 0). The nonlinear least squares fitting was implemented in the Bragg edge analysis. The fitting was performed for each 55 × 55 μm pixel of our dataset, thus requiring ~60,000 fits to be performed for each reconstructed image of parameters representing crystallographic properties of the samples, e.g. Figure 8. The results of the analysis for the reconstructed Bragg edge position are shown in Figure 8(a). There is substantial difference between the annealed and as-manufactured samples, as well as within the as-manufactured samples. The averaged *λ*_*e*_ values across the annealed sample was used as *λ*_0_ of the unstrained material, which was used to calculate the strain values shown in Figure 8(b). Substantial tensile residual strains are present in the non-annealed samples, with the highest strain concentration at the bottom (~1500 με) and at the tip (~2000 με) of the samples, as well as in the area around the center. The distribution of measured *λ*_*e*_ and strain values is shown in Figure 9, while the cross sections through the strain maps are shown in Figure 10. Figure 9 shows histograms of lattice spacing and strain distribution based on pixelated data over the entire samples. Note the distribution of *λ*_*e*_ is narrower for the annealed sample than for the as-built samples, which supports using post-printing annealing to produce an unstrained sample for strain analysis. Figure 9(b) shows that the strain distributions for both N1 and N2 are similar, about twice broader than the annealed sample, and both are in tensile, with maximum tensile strain up to ~1700 microstrain. Note that the measured strains are averaged over the sample thickness (5.2 mm along the Y-axis), and the transmission Bragg edge analysis shown here is sensitive *only* to the strain along the direction of neutron propagation (the Y-axis in this sample orientation).

Figure 10 shows the strain profiles along two specific directions, along the build direction Z and along the growth plane X. Each point represents strain, calculated for the spectrum integrated over 30 × 30 pixels (~1.6 × 1.6 mm^2^ area). The strain variations of the annealed sample is much smaller than those for N1 and N2, especially along the build direction (Figure 10(b)).

**Figure 11.**
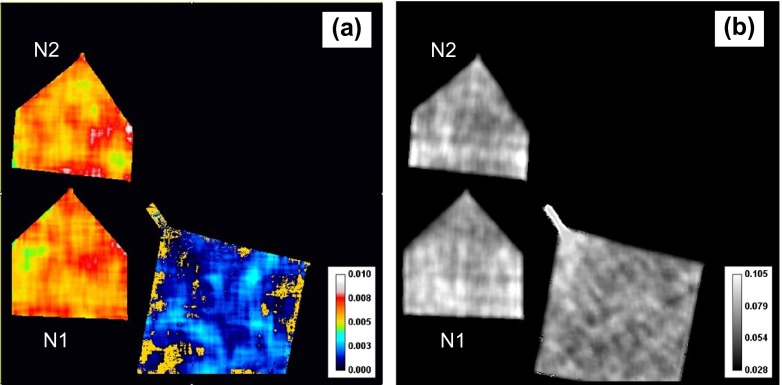
The maps of (2 0 0) Bragg edge parameters obtained by fitting Equation (3) to the measured data. (a) The width parameter *σ* (Å) calculated with 1.65 × 1.65 mm (30 × 30 pixels) running average over the area where spectra are obtained. (b) The height of the edge *C*_2_ calculated with 0.55 × 0.55 mm (10 × 10 pixels) running average of the spectra.

The maps of fitted parameters *σ* and *C*_2_ are shown in Figure 11. The width of the Bragg edge (Figure 11(a)) is a manifestation of grain size and intergranular defects distribution with respect to the neutron beam direction. For a single crystal sample which ideally is a single grain with a unique orientation, the measured spectrum shows a sharp dip as the Bragg scattering occurs at a single wavelength for a specific lattice plane. For a non-textured small grain polycrystalline sample for which grains are randomly oriented, the measured transmission has a smooth and gradual decrease from shorter wavelength towards the Bragg edge, appearing at the wavelength, defined by scattering from those grains whose lattice planes are perpendicular to the incident beam. For a relatively large size set of grains and for grains containing internal defects, the shape of the Bragg edge will appear different from these two extreme cases, and therefore neutron transmission spectra may be used as a qualitative measure of the degree of microstructure. A full spectrum Rietveld-type analysis is more appropriate for microstructural analysis and is not performed in this study as data analysis tools are still under development. The Bragg edge width *σ*, on the other hand, is a measure of edge sharpness and is related to the mosaic spread of lattice plane orientation perpendicular to the beam. As the curve fitting method (shown in Figure 7) covers only a limited wavelength range, *σ* doesn’t measure the overall microstructure of the sample. In Figure 11(a), the annealed sample shows overall narrower edge width than as-built samples, which probably means that annealing has reduced internal defects and increased the size of grains which are nearly aligned to the direction of the Y-axis. The consistency of edge width between N1 and N2 is indicative of the robustness of the curve fitting technique, as the mosaic spreads of grain orientations for as-built samples are expected to be similar. The maps of fitted height and the offset parameters of the Bragg edge (parameter *C*_2_ of Equation (3)) reveal the presence of some horizontal (along the growth planes) and vertical (along the growth direction) lines, caused by variation of the sample microstructure, namely the presence of referred grain orientations in this case. The height of the Bragg edge (parameter *C*_2_) gets smaller with the presence of preferred grain orientations, as seen in Figure 11(b). The striations along Z-axis seen in these images indicate that grain orientations have some mm-scale correlation between the layers along the build direction. These features are not seen in the narrow-energy image (Figure 5) acquired around the (2 0 0) Bragg edge, demonstrating the increased sensitivity of fitted data to the microstructure variation. The higher energy resolution of the pulsed neutron source is crucial for revealing such microstructural non-uniformities, which may not be possible with continuous neutron sources.

**Figure 12.**
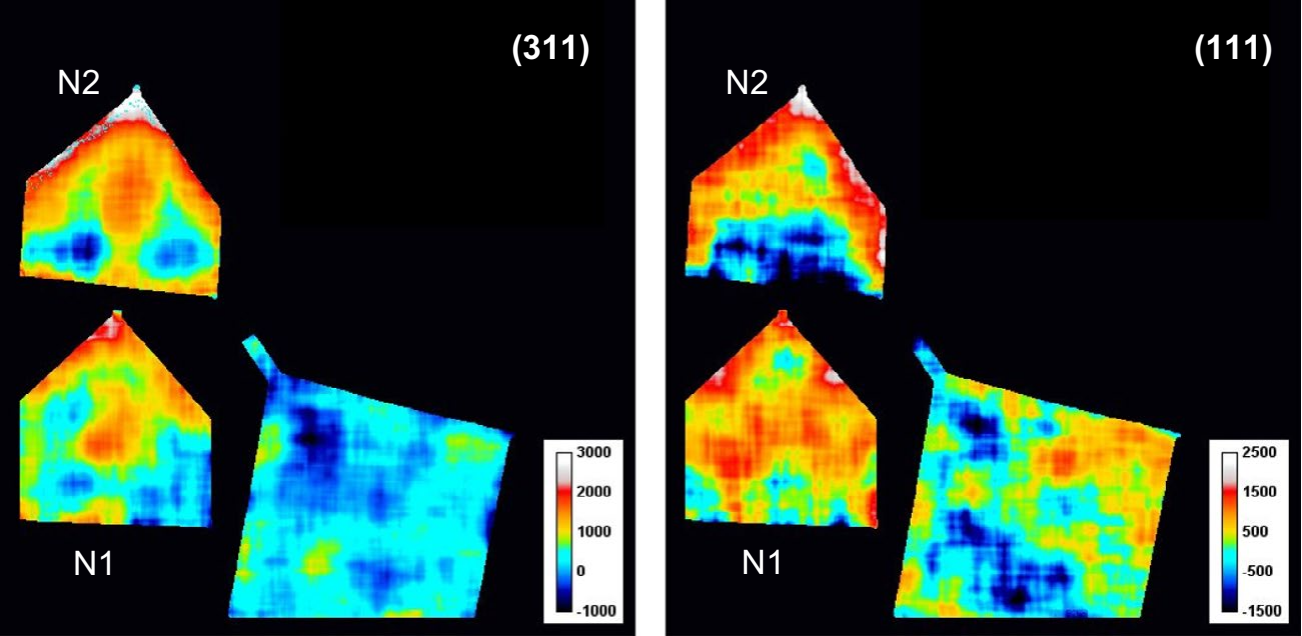
Strain maps along Y-axis measured for the (3 1 1) and (1 1 1) Bragg edges. Averaged spectra in the 1.65 × 1.65 mm area are used as in Figure 8, but accuracy of reconstructed strain for these edges is lower as these Bragg edges are not as strong as (2 0 0), leading to noisier fitted results. The average *λ*_0_ values from the annealed sample, 2.1655 Å and 4.1415 Å, respectively, were used as unstrained values for calculating the (3 1 1) and (1 1 1) strain maps.

**Figure 13.**
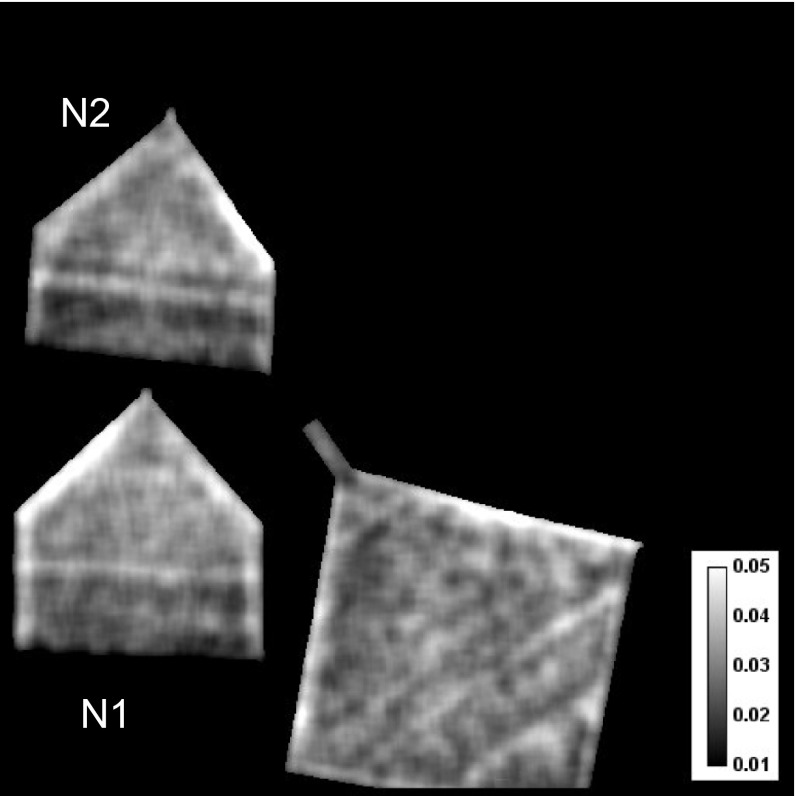
The maps of (3 1 1) Bragg edge height (parameter *C*_2_) obtained by fitting Equation (3) to the measured data. Fitted map of parameter *σ* is not shown as it was too noisy due to lower neutron counting statistics for that edge. The height of the edge *C*_2_ is calculated with 0.55 × 0.55 mm running average of the spectra.

The strain maps obtained from the Bragg edge analysis of (3 1 1) and (1 1 1) edges (Figure 12) show different residual strain values for different crystallographic planes. For example, the strain reconstructed from the (1 1 1) edge for sample N2 shows the compressive strains at the bottom of the sample, and nearly zero-strain values at the bottom of sample N1. Actually the strains from different edges are not necessarily the same as they are from different grains. For example, the (2 0 0) edge is formed by Bragg scattering from a set of grains whose (2 0 0) planes are perpendicular to the neutron beam, while the (1 1 1) edge is formed by a *different* set of grains whose (1 1 1) planes are perpendicular to the neutron beam. These two sets of grains with different orientations may have experienced different local stresses. The intergranular interaction may put one grain in tension and its neighboring grains in compression. Even when both grains are under the same stress, their strain responses will depend on Miller indices. Moreover there is a strong grain orientation level and distribution for different reflections as observed in Figures 11(b) and 13. For a Ni base alloy like Inconel 625, the (1 1 1) planes are harder to deform than (2 0 0) planes. In other words, under the same stress, one would expect a larger strain from the (2 0 0) edge than the (1 1 1) edge. Considering the multiplicity and the distribution of the grains along the three (1 1 1), (2 0 0) and (3 1 1) directions, for an estimation of the average strain in the sample it is more reliable to base it on the one exhibiting the higher spherical symmetry, as is the (3 1 1) reflection. The stresses experienced by certain grains result from a combination of thermal cycles and geometric constraints from the preceding layer onto which the new layer is formed and from their neighboring grains. Qualitatively, it is feasible to visualize that during heating or cooling of the manufacturing process, the thermal expansion can induce different strain responses from different grains depending upon their orientations; and due to their different elastic constants, some grains may be under tension and some under compression. The total stress might be balanced or cancelled out when stresses from all grains are taken into account.

Figure 13 shows the distributions of edge height (coefficient *C*_2_) for the (3 1 1) Bragg edge. Distinct horizontal features parallel to the growth planes are apparent for all three samples, with the most prominent line present in the middle of samples N1 and N2. Their connection to the manufacturing process is being investigated.

### 3.4. Strain and microstructure along X-axis (along the growth planes)

**Figure 14.**
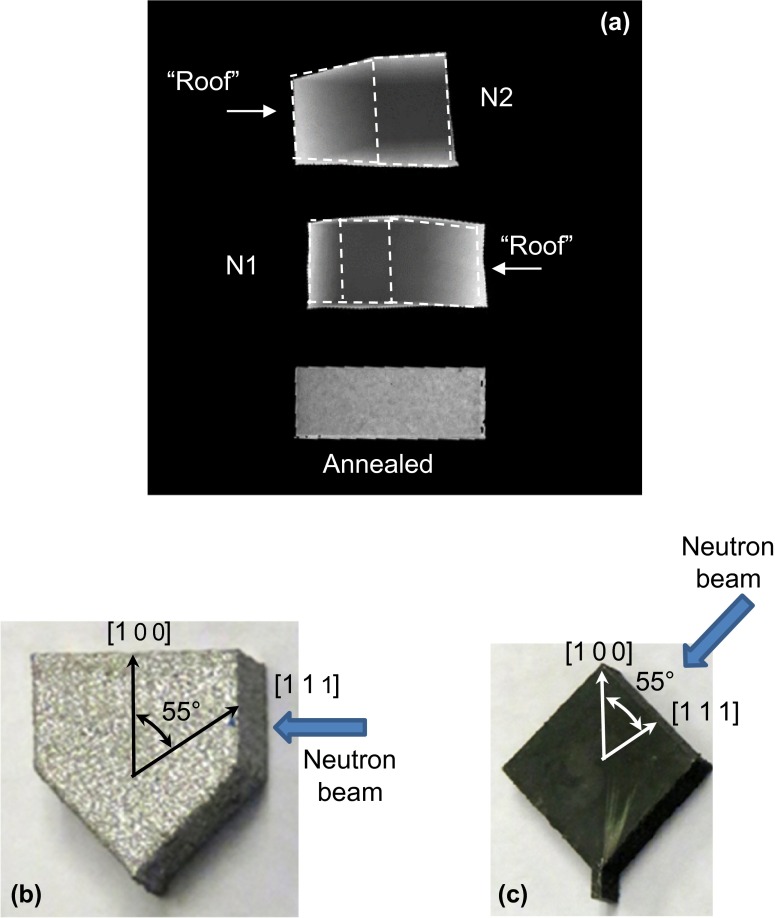
(a) Full spectrum transmission images obtained when the neutron beam entered the sample edge on. Samples N1 and N2 were oriented with neutrons travelling along the growth plane (along the X-axis, as shown in (b)), while for the annealed sample neutron beam was approximately perpendicular to an edge, or at 45° relative to the growth plane (as shown in (c)).

**Figure 15.**
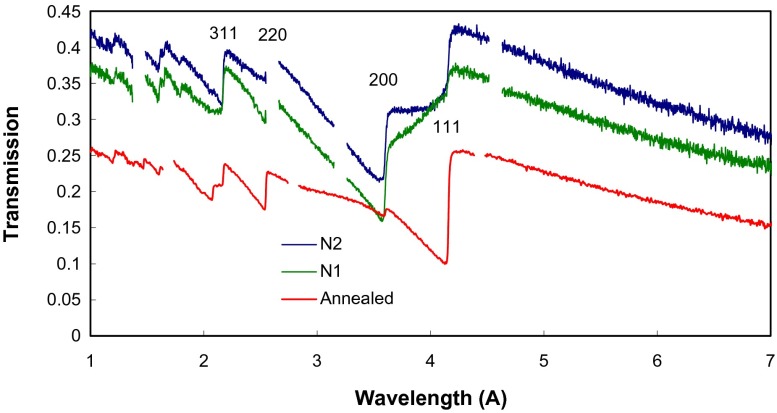
The measured transmission spectra, combined data from all the pixels within a sample, with neutron beam propagating along the growth plane (the X-axis) for samples N1 and N2 and at 45° relative to the growth plane for the annealed sample. The gaps in the spectrum are due to the detector deadtime required for the data readout. The pronounced presence of texture in sample N1 is evident by the slow transmission increase between (2 0 0) and (1 1 1) edges.

To understand the strain distribution in the orthogonal direction, the measurements were also performed with samples positioned in the edge-on orientation relative to the neutron beam. For the annealed sample, the neutron beam was at 45° with respect to the X-axis (i.e. perpendicular to one of the edges of the sample), while samples N1 and N2 were positioned such that neutrons propagate nearly parallel to their growth planes, along the X-axis. The white-beam neutron transmission radiography shown in Figure 14 exhibits no strong features. Not even pores or impurities which are seen in Figure 3 are visible in this sample orientation. The neutron transmission integrated across the entire thickness (~9 mm) of the sample might have canceled out the effect of small pores or impurities seen in Figure 3. However, the spectra shown in Figure 15 reveal substantial differences in the bulk microstructure of these samples. The (2 0 0) Bragg edge is nearly absent in the spectra measured for the annealed sample. At the same time, the sharpness of (1 1 1) edge for the annealed sample is substantially higher than that of samples N1 and N2. The small height of the (2 0 0) edge and sharpness of the (1 1 1) edge could be interpreted as that the build direction is along (1 0 0), which is the diagonal of this sample, which would result in large population of (1 1 1) and small populations of (2 0 0) planes perpendicular to the beam. Note the angle between (1 0 0) and (1 1 1) is 54.74° and this is very close to the incident beam direction. This is also consistent with the data from N1 and N2, as both samples exhibit a sharp (2 0 0) edge: this is because the neutron beam was along the X-axis and there would be a large population of grains with (2 0 0) planes perpendicular to the beam if the build direction is along (1 0 0). For the same reason, there would be a small population of (1 1 1) planes perpendicular to the neutron beam for N1 and N2; as a result, the (1 1 1) edge is nearly missing for N1.

**Figure 16.**
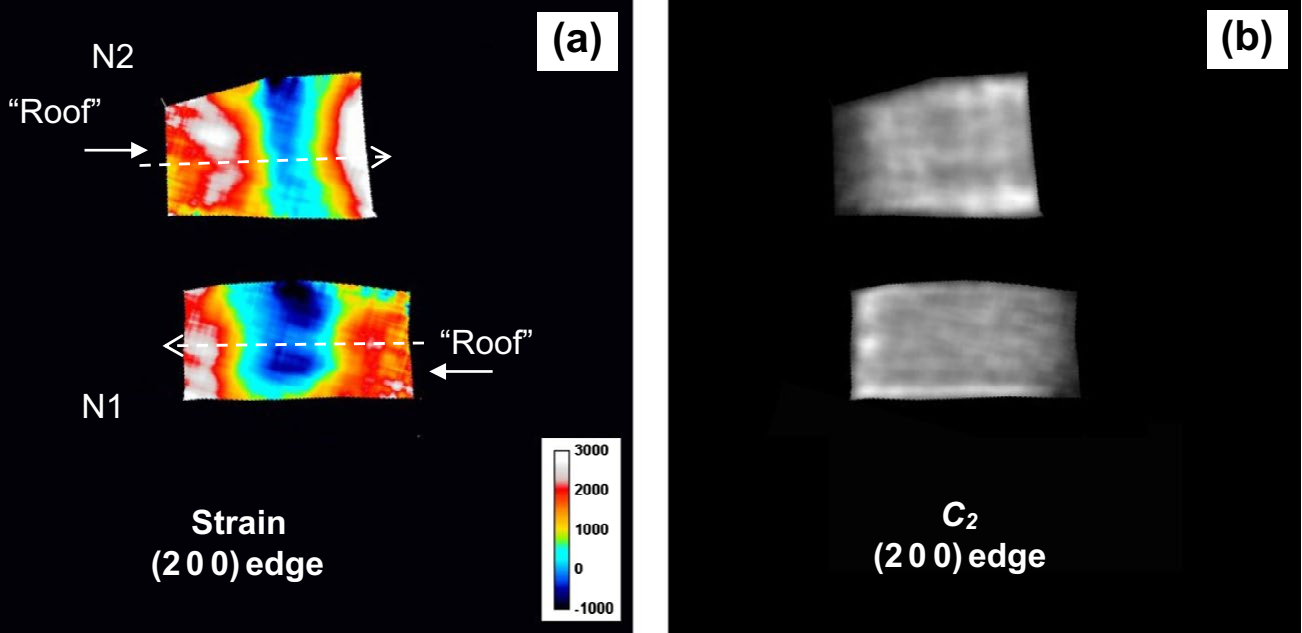
(a) The map of averaged strain along the X-axis calculated by fitting to measured neutron transmission spectra of (2 0 0) Bragg edge. (b) The edge height parameter *C*_2_ measured when samples N1 and N2 were mounted with neutrons travelling along the growth plane (X-axis). The strain was obtained with 1.65 × 1.65 mm running average of the spectra, while the parameter C_2_ was obtained with 0.55 × 0.55 mm averaging of the spectra. The dashed lines in (a) indicate the location of cross section through the strain map shown in Figure 17.

**Figure 17.**
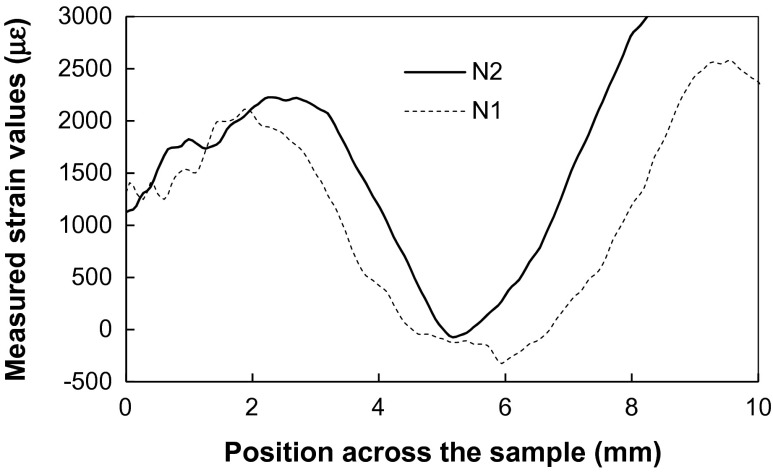
Cross sections (55 μm wide) through the X-axis strain maps for samples N1 and N2 calculated for (2 0 0) Bragg edge shown in Figure 16(a).

The strain map and the map of the (2 0 0) Bragg edge height parameter *C*_2_ for the samples N1 and N2, shown in Figure 16, indicate that the tensile strain is present at the top and at the bottom of these samples, similar to the strains averaged along Y-axis. However, the middle section of these samples did not show tensile strain along the X-axis, but rather small compressive strain values, as shown in Figure 17. There are also some features parallel to the growth direction seen in Figure 16(b), confirming the above-mentioned correlation of grain orientations between subsequent layers. However, that correlation is not preserved along the Y-axis as the layers along that axis are formed by separate passes during sample manufacturing.

**Figure 18.**
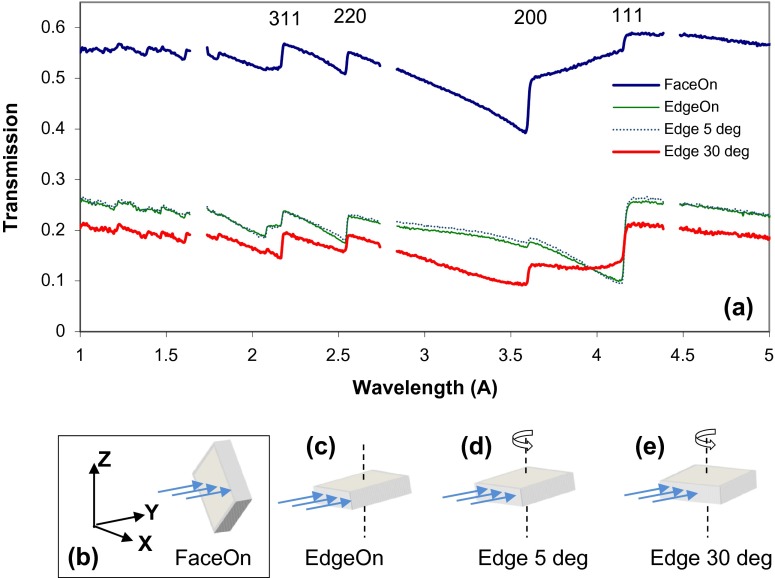
The transmission spectra of the annealed sample measured at different sample orientations relative to the incoming beam. The sample build direction is along the Z-axis (b). The sample orientations are as follows: (b) Face-on – with neutron incidence along Y-axis, (c) Edge on – 45° relative to the sample growth plane (X-axis); (d) and (e) the sample was rotated by 5 and 30° around the Y-axis, respectively.

To study the variation of microstructure dependence on sample orientation, the annealed sample was measured in the edge-on orientation with 0, 5 and 30° rotations around the Y-axis, Figure 18. The transmission spectra of the annealed sample showed substantial variation with sample rotation, indicating the presence of strong texture of (1 0 0) as build direction. The height of (2 0 0) edge, for example, is larger for the neurons propagating along Y-axis and X-axis (orientations (b) and (e) in Figure 18), but is smaller when neutrons propagate at 45° relative to the X-axis (orientations (c) and (d)). At the same time, the (1 1 1) edge is the strongest for the orientations (c) and (d) and weakest for orientation (b). All these observations can be explained by the assumption that the sample is (1 0 0) textured in the build direction.

## 4. Conclusions

The results of our experiments with Inconel 625 samples, manufactured by a direct metal laser melting method, demonstrate the possibilities of using energy-resolved neutron imaging to study bulk microstructure properties of additive manufactured components. The low attenuation of neutrons for various metals provides the possibility to study thick samples non-destructively, when it is impossible with other characterization techniques. The contrast in energy-resolved neutron imaging due to neutron coherent scattering can reveal unique crystallographic properties of the samples, most importantly the distribution of internal strains and stresses (averaged over the neutron path through the sample), the grain size and intra-granular defects distribution, and the presence of texture or preferred orientation of crystalline grains. The spatial resolution of this imaging technique is currently determined by the resolution of event counting detectors (55 μm in our case), the neutron beam intensity and the data integration time. The distribution of macroscopic (~100 μm) defects such as voids and impurities can also be imaged through the entire volume of the samples along with strain and microstructure. At the same time the elemental distribution within the sample may be obtained with resonance absorption imaging,[19,20] providing the elements of interest have reasonably high resonance absorption cross section, such as Ta, W and Hf which are commonly in Ni-base alloys. As very high neutron flux is essential for this measurement, such measurements cannot be accomplished by using portable neutron sources which may be desirable for process or quality control. This technique obviously cannot be applied for real-time feedback control of manufacturing processes as it can be implemented only in large scale neutron beamline facilities. However, it can be an additional tool in the optimization of specific additive manufacturing techniques and may be used to correlate spatial variation of microstructures with processing parameters of a specific manufacturing method and materials composition.

## Disclosure statement

No potential conflict of interest was reported by the authors.

## Funding

This work was supported in part by the US Department of Energy [STTR grant numbers DE-FG02–07ER86322, DE-FG02–08ER86353 and DE-SC0009657].
